# Water Soluble PMPC-Derived Bright Fluorescent Nitrogen/Phosphorous-Doped Carbon Dots for Fluorescent Ink (Anti-Counterfeiting) and Cellular Multicolor Imaging

**DOI:** 10.3390/polym15061352

**Published:** 2023-03-08

**Authors:** Suguna Perumal, Raji Atchudan, Thomas Nesakumar Jebakumar Immanuel Edison, Sambasivam Sangaraju, Weslen Vedakumari Sathyaraj, Yong Rok Lee

**Affiliations:** 1 Department of Chemistry, Sejong University, Seoul 143747, Republic of Korea; 2 School of Chemical Engineering, Yeungnam University, Gyeongsan 38541, Republic of Korea; 3 Department of Chemistry, Saveetha School of Engineering, Saveetha Institute of Medical and Technical Sciences, Chennai 602105, Tamil Nadu, India; 4 Department of Chemistry, Sethu Institute of Technology, Kariapatti 626115, Tamil Nadu, India; 5 National Water and Energy Center, United Arab Emirates University, Al Ain 15551, United Arab Emirates; 6 Faculty of Allied Health Sciences, Chettinad Hospital and Research Institute, Chettinad Academy of Research and Education, Kelambakkam 603103, Tamil Nadu, India

**Keywords:** PMPC, hydrothermal, carbon dot, fluorescent ink, anti-counterfeiting, cellular multicolor imaging

## Abstract

Here, a simple one-step hydrothermal-assisted carbonization process was adopted for the preparation of nitrogen/phosphorous-doped carbon dots from a water-soluble polymer, poly 2-(methacryloyloxy)ethyl phosphorylcholine (PMPC). By the free-radical polymerization method, PMPC was synthesized using 2-(methacryloyloxy)ethyl phosphorylcholine (MPC) and 4,4′-azobis (4-cyanovaleric acid). The water-soluble polymers, PMPC, that have nitrogen/phosphorus moieties are used to prepare carbon dots (P-CDs). The resulting P-CDs were thoroughly characterized by various analytical techniques such as field emission-scanning electron microscopy (FESEM) with energy-dispersive X-ray spectroscopy (EDS), high-resolution transmittance electron microscopy (HRTEM), X-ray diffraction (XRD), Raman spectroscopy, attenuated total reflectance Fourier transform infrared (ATR-FTIR) spectroscopy, X-ray photoelectron spectroscopy (XPS), Ultraviolet-visible (UV-vis) spectroscopy and fluorescence spectroscopy to determine their structural and optical properties. The synthesized P-CDs displayed bright/durable fluorescence, were stable for long periods, and confirmed the enrichment of functionalities including oxygen, phosphorus, and nitrogen heteroatoms in the carbon matrix. Since the synthesized P-CDs showed bright fluorescence with excellent photostability, excitation-dependent fluorescence emission, and excellent quantum yield (23%), it has been explored as a fluorescent (security) ink for drawing and writing (anti-counterfeiting). Further, cytotoxicity study results advised for biocompatibility and thus were used for cellular multicolor imaging in nematodes. This work not only demonstrated the preparation of CDs from polymers that can be used as advanced fluorescence ink, a bioimaging agent for anti-counterfeiting, and cellular multicolor imaging candidate, but additionally prominently opened a new perspective on the bulk preparation of CDs simply and efficiently for various applications.

## 1. Introduction

Carbon dots (CDs) are 0-dimensional carbon materials that have a size less than 10 nm, and have been considered recently [1,2,3]. CDs are composed of sp^2^/sp^3^ hybridized carbon atoms [1,2,3]. CDs have exhibited unique properties such as high water dispersibility, tunable optical properties, flexibility surface modification, and biocompatibility [4,5]. These properties exist in the CDs because of the functional groups such as carbonyl, hydroxyl, amine, carboxylic acid, amide, and so on [4,5]. Moreover, the existence of the functional groups plays an important role in the optical properties. The surface functional groups of synthesized CDs can be tuned with different heteroatoms using different carbon sources that are enriched with different heteroatoms [6,7]. CDs find applications in a wide range, including in catalysis for improving air pollution, such as photocatalysis [8,9,10]. The turn-off and turn-on properties of CDs proposed the use of CDs in detecting heavy metal ions and small molecules and dyes in polluted water [11,12,13]. CDs are employed as electrocatalysts for the evolution of hydrogen, oxygen, carbon reduction, supercapacitors, and batteries [14,15]. The biocompatibility properties of CDs encourage their use in many bioapplications including bioimaging, biosensing, and drug delivery [16,17]. Due to the high quantum yield, water-solubility, photostability, and ease of washing CDs, CDs are used as fluorescent ink [18,19].

CDs are prepared using either top-down or bottom-up methods including laser ablation [20], ultrasonication [21,22], arc discharge [23], simple heating [24], and solvothermal [25,26]. Chemical precursors such as acid derivatives, thiourea derivatives, and carbohydrates were employed to prepare CDs [27,28,29]. Recently much effort has been made in the preparation of CDs from biowaste [30,31] and plant sources [32,33]. Apart from the small molecules, polymers are employed as carbon sources for the synthesis of CDs [34,35,36,37,38,39]. Any functional group that is present in small molecules or polymers will be retained as surface functional groups in CDs [40,41,42]. Thus, the existence of a higher concentration of functional groups in CDs may alter the optical properties. As a result, CDs from polymers or small molecules show improved optical properties and high quantum yield compared to CDs obtained from plant sources [39,43,44]. This high quantum yield and optical properties play a vital role in applications such as fluorescent ink, bioimaging, sensing, and so on.

Thus, we utilized poly 2-(methacryloyloxy)ethyl phosphorylcholine (PMPC) as a carbon source for the synthesis of CDs. This polymer was prepared from the 2-(methacryloyloxy)ethyl phosphorylcholine monomer by free radical polymerization. PMPC is a biocompatible polymer and is used widely in bioapplication, enriched with nitrogen, phosphorus, and oxygen [45,46]. Since PMPC has heteroatoms it was believed that CDs from this polymer will exhibit excellent optical properties with low toxicity. Carbon dots from PMPC (P-CDs) were prepared using a simple hydrothermal method. As expected, the resulting P-CDs showed good optical properties with excellent stability and were suggested for the bioapplication. The prepared P-CDs were carefully characterized using field emission-scanning electron microscopy (FESEM) with energy-dispersive X-ray spectroscopy (EDS), high-resolution transmittance electron microscopy (HRTEM), X-ray diffraction (XRD), Raman spectroscopy, attenuated total reflectance Fourier transform infrared (ATR-FTIR) spectroscopy, X-ray photoelectron spectroscopy (XPS), Ultraviolet-visible (UV-vis) spectroscopy and fluorescence spectroscopy. After confirmation of the formation of P-CDs and their chemical composition, they were utilized as fluorescent ink for drawing, writing, and as a fingerprint tracer. Moreover, by confirming the low toxicity of P-CDs, they were employed as a bioimaging agent for nematode cells.

## 2. Materials and Methods

### 2.1. Materials

The 2-(methacryloyloxy)ethyl phosphorylcholine (MPC) (97%), S-basal buffer, sodium azide (NaN_3_), and 3-(4,5-Dimethylthiazol-2-yl)-2,5-diphenyltetrazolium bromide (MTT) were purchased from Sigma-Aldrich, Republic of Korea and used as received. 4,4′-Azobis(4-cyanovaleric acid) (ACVA) was purchased from Merck, Republic of Korea, and used as received. Distilled water was used throughout this study.

### 2.2. Synthesis of Poly 2-(methacryloyloxy)ethyl Phosphorylcholine

**Scheme 1:**
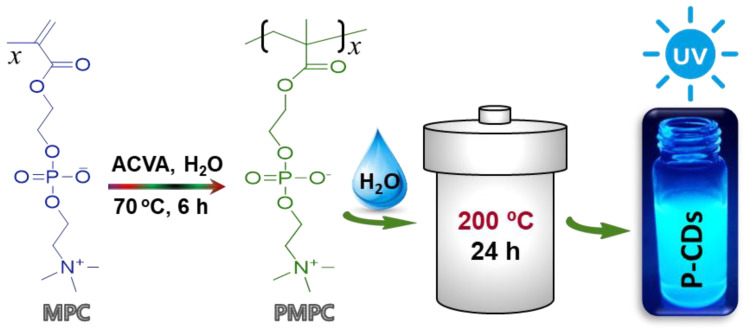
Synthesis of P-CDs from PMPC by one-step hydrothermal-assisted carbonization process.

PMPC was prepared as shown in Scheme 1. To 3.38 mmol of MPC in 70 mL, 0.16 mmol of ACVA was added in 200 mL of degassed water. The solution was further purged with nitrogen gas for 30 min and the reaction vessel was sealed well. Then, the solution was sonicated at 70 °C for 6 h, and after 6 h the reaction mixture was dialyzed in water for 3 days using a cellulose membrane with 3 k molecular weight. Then, the solid white powder was obtained by lyophilization of the dialyzed reaction mixture. The number molecular weight of the prepared PMPC was measured as 57,520 g/mol, with PDI as 2.52 using size-exclusion chromatography.

### 2.3. Synthesis of Nitrogen/Phosphorous-Doped Carbon Dots

Nitrogen/phosphorous-doped carbon dots were prepared hydrothermally using the above-synthesized PMPC. Typically, 250 mg of PMPC was dissolved in 50 mL of distilled water and then transferred into a Teflon-lined stainless steel autoclave with a capacity of 100 mL. The tightly closed autoclave was heated at 200 °C for 24 in a hot air oven to complete the formation of carbonized products. Post-completion of the hydrothermal carbonization, the autoclave was naturally cooled down to room temperature and dried to obtain a solid product by using a freeze drier. The resulting product was denoted as polymer-derived carbon dots (P-CDs) and the corresponding detailed synthesis route is shown in Scheme 1. The final P-CDs were utilized for further structural, optical, and biological analyses.

## 3. Results and Discussion

### 3.1. Structural Studies of Synthesized P-CDs

**Figure 1:**
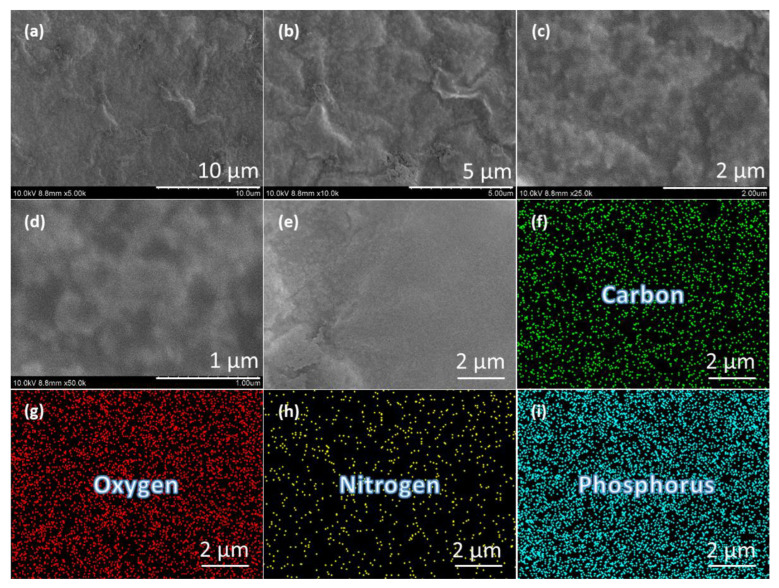
(**a**–**d**) FESEM images with different magnifications of P-CDs synthesized from PMPC; (**e**) FESEM electron image and the corresponding EDX mapping of (**f**) carbon, (**g**) oxygen, (**h**) nitrogen and (**i**) phosphorus elements of synthesized P-CDs.

**Figure 2:**
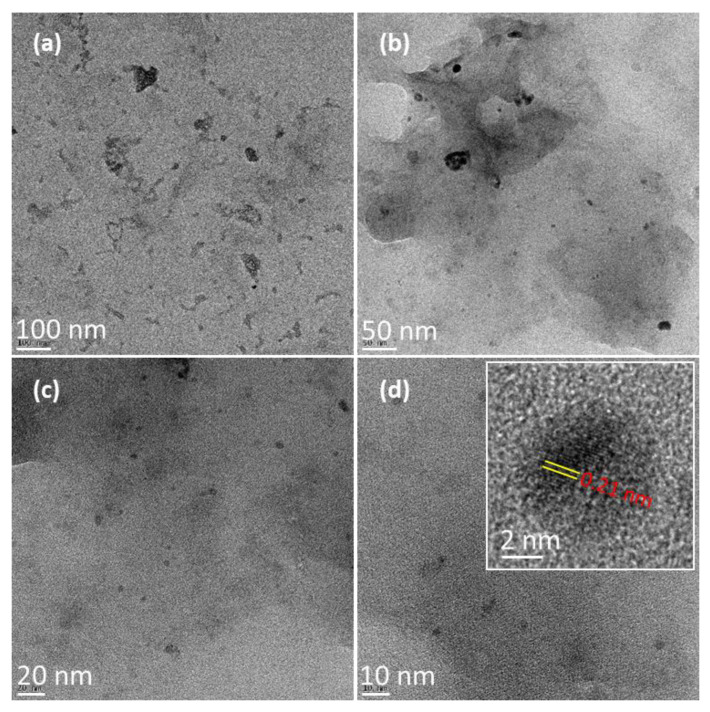
(**a**–**d**) TEM/HRTEM images with different magnifications of P-CDs synthesized from PMPC.

The prepared PMPC by free radical polymerization was utilized to prepare P-CDs by the hydrothermal method. The prepared PMPC was confirmed using a nuclear magnetic resonance spectrum (NMR) that measured 500 MHz (Figure S1). The broad peaks (g) between 0.8 and 1.2 ppm are responsible for the methyl group. A broad peak (f) around 2 ppm is attributed to the backbone of PMPC. The presence of three methyl groups from quaternary ammonium groups confirms a sharp peak (e) around 3.2 ppm. –CH_2_ attached to quaternary ammonium groups shows a peak (d) between 3.6 and 3.8 ppm. –CH_2_ attached to oxygen atoms of phosphate (c and d) and methacrylate (a) groups appeared between 4 and 4.4 ppm. The absence of impurity peaks other than the peaks that are responsible for the functional group that exists in PMPC confirms the purity of PMPC. P-CDs were characterized by several techniques to confirm their structural and chemical compositions. The surface morphologies of the P-CDs were examined using FESEM and TEM. The morphology studies using FESEM at different magnifications are shown in Figure 1. As observed from the FESEM images (Figure 1a–e), the P-CDs result in smooth surfaces. The existence of the smooth surface is of the aggregation of small-size P-CDs. EDX mapping (Figure 1f–i) strongly indicates that the synthesized P-CDs are successfully loaded with elements such as carbon (C), nitrogen (N), oxygen (O) and phosphate (P). The existence of these elements confirms that P-CDs are formed by the decomposition of PMPC. Further, the distributions of C, N, O, and P are uniform. Additionally, the ratios of elements of C, N, O, and P were obtained from EDX spectral results, which were 61, 28, 7, and 4, respectively. The measurement of the size of P-CDs exceeds because of the resolution of the FESEM, which suggests that the TEM measurement has high-resolution power. Thus, the prepared P-CDs were observed using TEM. Figure 2 shows that the TEM images of P-CDs reveal spherical shaped P-CDs. The average diameter of P-CDs was estimated as ~5 nm by randomly counting the particle size on the HRTEM images (Figure 2a–c). The inset of Figure 2d (HRTEM) displays the crystalline structure with a lattice spacing of about 0.21 nm corresponding to the graphite (100) plane [47].

X-ray diffraction patterns reveal the crystallinity of a material. The powder XRD pattern of P-CDs (Figure S2) depicts a broad peak centered at 20° corresponding to the (002) lattice, which indicates the presence of disordered carbon atoms with amorphous nature due to the presence of many functional groups [48,49]. Moreover, the broad diffraction peak (2θ = 20°) of synthesized P-CDs reveals that the synthesized carbon particles are smaller in nature. The size of the P-CDs was calculated as 4.5 nm using an XRD pattern ((0 0 2) plane), which shows a consistent size with that measured using HRTEM. In addition, five sharp peaks at 16.8, 23.9, 29.2, 33.9 and 45.2° show the crystalline nature of the materials. This is believed to be because of the presence of heteroatoms in P-CDs [50]. Figure S3 shows the Raman spectrum of the synthesized P-CDs. The Raman spectrum in Figure S3a exhibits a noisy peak centered at ~1560 cm^−1^ (Raman wavelength ranges from 750 to 1750 cm^−1^). Noticeably, there is no clear separation for D- and G-bands in the Raman spectrum of synthesized P-CDs. These peaks overlapping might be due to the wealthy functional groups accumulated on the surface of the synthesized P-CDs. However, Figure S3b shows two peaks on deconvolution that correspond to D- and G-bands positioned at 1170 and 1500 cm^−1^, respectively. The presence of D- and G-bands confirms the presence of sp^2^ carbon and sp^3^ defects, respectively [51,52]. The intensity of the D-band is less than the G-band (I_D_ < I_G_), indicating that the P-CDs are well graphitized. The I_D_/I_G_ ratio for the synthesized P-CDs was calculated as 0.6, and this suggests that the carbon structures present in P-CDs have a smaller amount of defects [52].

**Figure 3:**
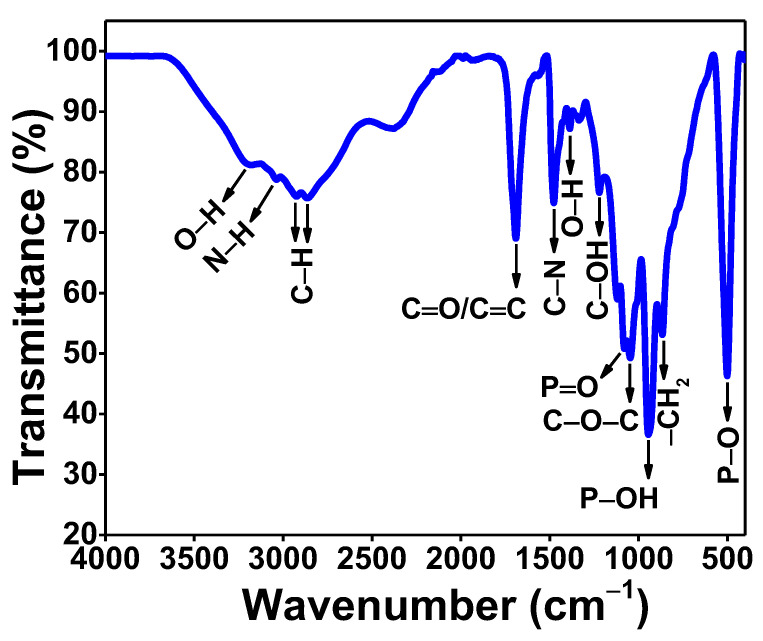
ATR-FTIR spectrum of P-CDs synthesized from PMPC.

Figure 3 depicts the FTIR spectrum of P-CDs; stretching vibrations of O–H, N–H, asymmetric C–H and symmetric C–H are observed at ~3215, 3050, 2840, and 2950 cm^−1^, respectively. The presence of strong absorption peaks at 1695 and 1480 cm^−1^ are revealed for C=O/C=C and C–N groups, respectively. The presence of the C–N–C functional group confirms that the nitrogen atoms bonded with carbon atoms, which means they chemically bonded with the carbon matrix. The bending vibration of O–H was observed at 1386 cm^−1^. A stretching vibration of C–OH at 1228 cm^−1^, stretching vibration of P=O/C–O–C at 1100–1030 cm^−1^, and strong bands for the stretching vibration of P–OH at 948 cm^−1^ were observed. The strong absorption band at 506 cm^−1^ was attributed to P–O stretching [53]. These results reveal that there are abundant functional groups such as C=O, O–H, N–H, C=C/C=O, C–N, P=O, C–O–C, P–OH and P–O. These presented hydrophilic functional groups make the P-CDs have excellent water dispersibility, which reorganizes their preferred modifications to diverse applications.

**Figure 4:**
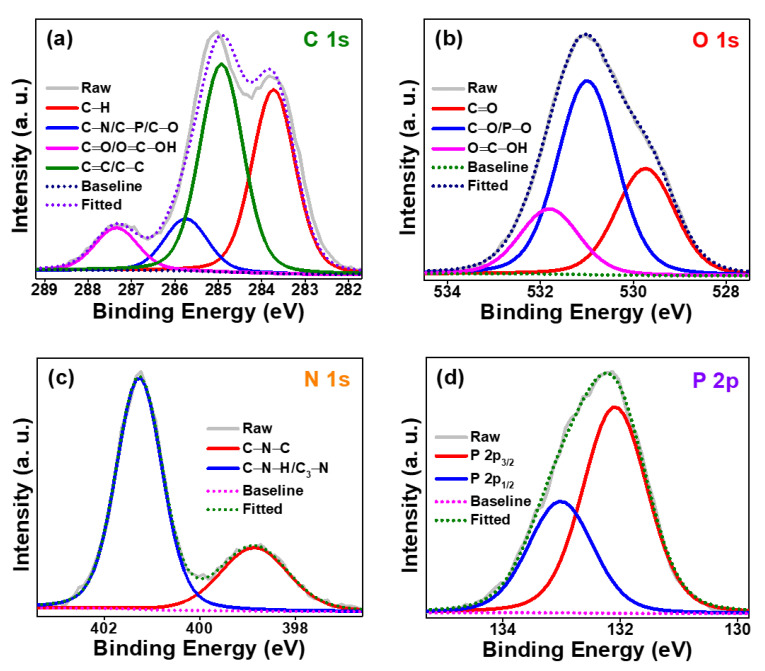
XPS spectra of P-CDs synthesized from PMPC. High resolution XPS of P-CDs with deconvolution of (**a**) C 1s, (**b**) O 1s, (**c**) N 1s and (**d**) P 2p levels.

Further, the chemical composition of P-CDs was confirmed from XPS measurements. Figure S4 reveals the presence of C, N, O and P. The XPS spectrum of P-CDs shows two strong and three weak peaks at 132 (P 2p), 190 (P 2s), 285 (C1s), 401 (N 1s) and 531 eV (O 1s). The inset of Figure S4 shows the atomic percentages of C, O, N and P as 57, 30, 8 and 5%, respectively. These values are comparable with those obtained using EDX from FESEM. Figure 4a shows that the high resolution of C 1s exhibits four peaks at 283.7, 284.9, 285.7 and 287.3 eV that are associated with C–H, C=C/C–C, C–N/C–P/C–O and C=O/O=C–OH, respectively [54]. The deconvolution of the O 1s (Figure 4b) displays three peaks at 529.7, 531.0 and 531.8 eV, suggesting the presence of C=O, C–O/P–O, and O=C–OH groups, respectively. The N 1s spectrum of P-CDs (Figure 4c) shows two prominent peaks at 398.9 and 401.2 eV, which can be attributed to C–N–C and C–N–H/C_3_–N, respectively. The high-resolution P 2p spectra can be deconvoluted into two peaks, namely P 2p_3/2_ (132.1 eV) and P 2p_1/2_ (133.0 eV). The presence of functional groups such as C–H, C–N/C–P/C–O, C=O, O=C–OH, C=C/C–C, C–N–C, C–N–H/C_3_–N in the synthesized P-CDs is confirmed form FTIR and XPS studies. These functional groups are expected to play an important role in water dispersibility, optical properties and biocompatibility.

### 3.2. Optical Studies of Synthesized P-CDs

**Figure 5:**
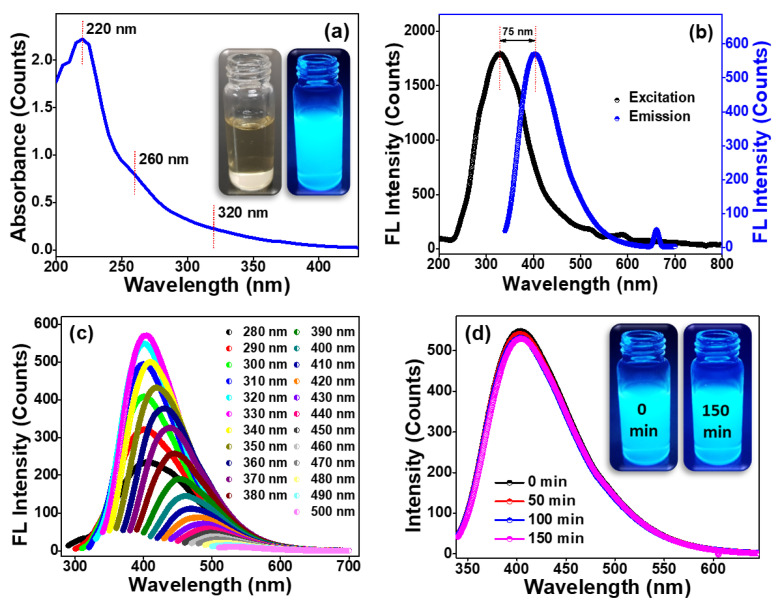
(**a**) UV–Vis absorption spectrum of the synthesized P-CDs. Inset (**a**): Digital photographic image of the P-CDs aqueous dispersion under visible light (left) and UV light (right). (**b**) Fluorescence excitation and emission spectra of synthesized P-CDs. (**c**) Fluorescence emission spectra of synthesized P-CDs at excitation wavelength from 280 to 500 nm in 10 nm increments. (**d**) Fluorescence emission spectra of synthesized P-CDs at UV irradiation time from 0 to 150 min in 50 min intervals. Inset (**d**): Digital photographic image of the P-CDs aqueous dispersion under UV light (365 nm) at UV irradiation time 0 min (left) and 150 min (right).

The optical properties of P-CDs were studied using UV-Vis and fluorescence spectroscopy. The UV-Vis spectrum of the water-diluted solution from P-CDs (Figure 5a) shows three absorption peaks at 220, 260 and 320 nm. The strong peak at 220 nm is due to the π − π* transition of the C=C from the sp^2^ carbon of P-CDs [55,56]. The shoulder peak at 260 nm is ascribed to the n − π* transition from the functional groups such as C=O [56,57]. The shoulder peak around 320 nm is responsible for exciting surface defects because of the presence of hetero atoms in P-CDs [58,59]. An enlarged UV-Vis spectrum of P-CDs (Figure 5a) between the range of 240–420 nm was provided for clear observation of the shoulder peak (320 nm), shown in Figure S5. The inset of Figure 5a shows the photographic images of diluted samples of P-CDs in water under visible light (left) and UV light (right). The colorless solution under visible light turns to bright blue fluorescence under UV light.

The fluorescence of the P-CDs is associated with surface defects that were confirmed by XRD and Raman analyses. However, P-CDs show fewer surface defects, and thus it was expected to have a better fluorescence performance [52,60]. The fluorescence spectra of P-CDs exhibited excitation and emission peaks at 330 and 400 nm, respectively (Figure 5b). As shown in the fluorescence excitation and emission, a shift of 75 nm was observed between the excitation and emission wavelengths. The Stoke shift is the difference between the energy of the absorbed and released photon. Figure 5c depicts the fluorescence emission spectrum; when P-CDs are excited with different wavelengths they emit light at different wavelengths with different intensities. On excitation between 280 and 500 nm with an increase of 10 nm, P-CDs show emission at every wavelength. The emission intensity increases from excited wavelength 280 to 330 nm. A high emission intensity is observed at an excitation wavelength of 330 nm, with an excellent quantum yield of 23%. After this excitation wavelength, the emission intensity decreased. Additionally, the excitation wavelength gradually increased from 280 to 500 nm, and the emission peak position of P-CDs became redshifted from 390 to 535 nm. The excitation-dependent was due to the minor intersection between the excitonic absorption and emission of the material; this favorable for efficient fluorescence emission [61]. The stability of fluorescence is very important for the application. Thus, stability was examined by the continuous irradiation of the P-CDs solution with UV light for 150 min. Figure 5d reveals that the fluorescence intensity remains unchanged even after 150 min irradiation of UV light at 365 nm. Further, the solutions of P-CDs with 0 and 150 min irradiation of UV light remain the same (Figure 5d inset). This confirms the stable fluorescence properties of the synthesized P-CDs.

Generally, the fluorescence spectra of P-CDs are normalized to show the excitation at different wavelengths. Figure S6 depicts the normalized fluorescence intensity obtained for P-CDs at different wavelengths. Normalized spectra disclose the redshift of the intensity with an increase in the excitation wavelengths. This shift might account for the existence of several functional groups [62] in P-CDs that are confirmed by FTIR and XPS studies.

### 3.3. Fluorescent Ink and Anti-Counterfeiting Studies of Synthesized P-CDs

**Figure 6:**
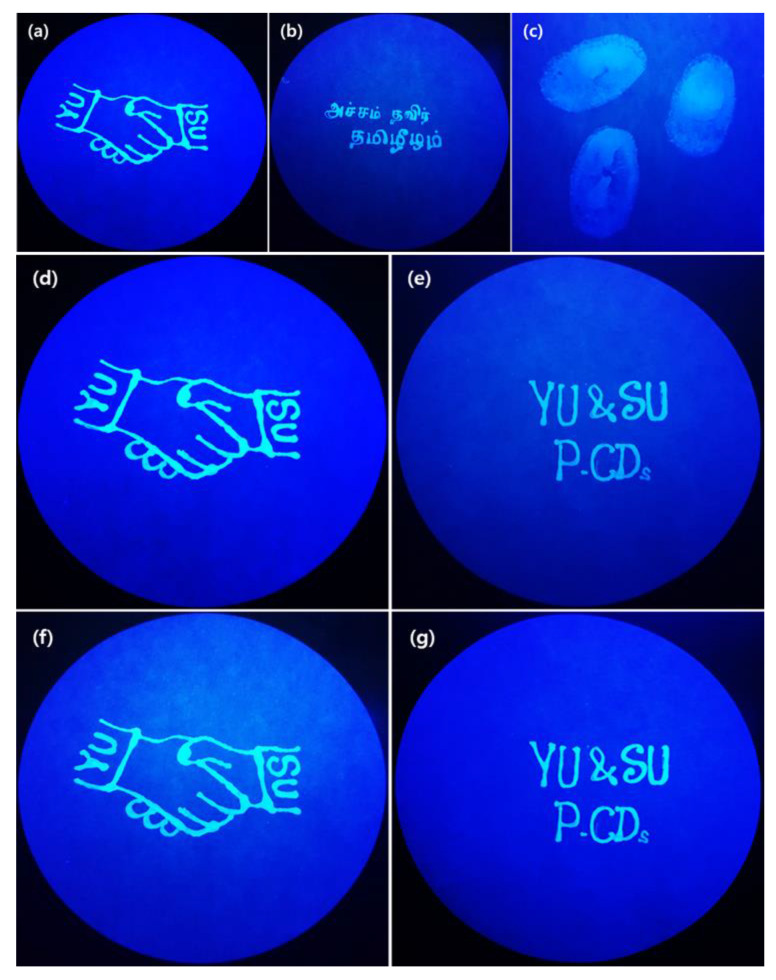
Digital photographic images of P-CDs ink-loaded commercial Whatman filter paper under 365 nm UV light; (**a**) hand-drawn pattern image, (**b**) handwritten words, and (**c**) P-CDs-formed fingerprints; (**d**) hand-drawn pattern and (**e**) handwritten letter images at the time of drawing/writing and the corresponding (**f**) hand-drawing pattern and (**g**) handwritten letter images after 4 months.

The P-CDs were further employed as fluorescent ink based on their high dispersibility in water and stable photostability. The aqueous solution of P-CDs was injected in the writing pen and facilitated for drawing pictures, as shown in Figure 6a, for writing text, as shown in Figure 6b, and for tracing fingerprints, as shown in Figure 6c, on the Whatman filter paper. The drawing, text, and fingerprints were visible under UV light with an excitation wavelength of 365 nm. This suggests that the synthesized P-CDs can be a promising candidate in many applications, including secret file labeling, printing stamps, ink pads for fingerprints, and so on.

Moreover, the stability of the pattern application was examined by storing the hand-drawing pattern image and text for a long time. The hand-drawing pattern image and handwriting text on Whatman filter paper shown in Figure 6d,e were obtained immediately after drawing and writing and were stored for 4 months. After the storage time, the fluorescence intensity of the hand-drawn pattern image and handwritten text shows an insignificant change (Figure 6f,g). Further, the handwritten letters and hand-drawn patterns are easily washable with water. This suggests that P-CDs can be used in practical applications because they can be simply washed out with water, have stable fluorescence intensity, and are eco-friendly and cost-effective compared to commercially available dye agents and polymer composites that are utilized in pattern applications [61,63,64,65].

### 3.4. Biological Studies of Synthesized P-CDs

Using many techniques, the synthesized P-CDs were studied well and the presence of many functional groups such as C=O, C=C, O–H, N–H, C–N and C–N–H/C_3_–N was confirmed. The presence of these functional groups plays an important role in the optical properties. Thus, it was expected that the prepared P-CDs will be a promising bioimaging agent.

**Figure 7:**
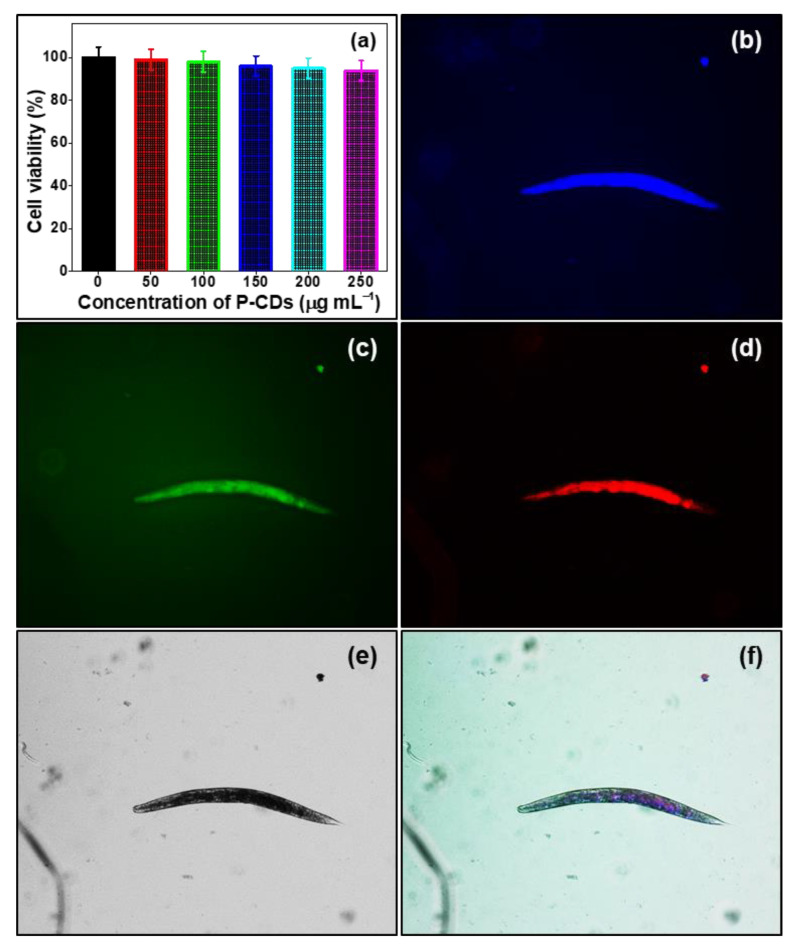
(**a**) Cell viability values (%) estimated by MTT assay versus incubation concentrations of P-CDs (0, 50, 100, 150, 200 and 250 μg mL^−1^) at 25 °C for 48 h. The confocal fluorescence microscopy images of nematodes (Caenorhabditis elegans) treated with 100 μg mL^−1^ P-CDs for 24 h under excitation of (**b**) 400 nm, (**c**) 470 nm, (**d**) 550 nm, (**e**) bright-field and (**f**) overlap. Live Caenorhabditis elegans were immobilized using 0.05% NaN_3_ for imaging under fluorescence filters.

To evaluate the bioimaging application of P-CDs, the cytotoxicity of P-CDs was performed with an MTT assay. The nematode cells were cultivated with different concentrations of P-CDs between 0 and 250 μg mL^−1^ for 48 h, without P-CDs (0 μg mL^−1^) used as control. The cell viability of nematodes with and without P-CDs is shown in Figure 7a. As shown in Figure 7a, nematode cell survival percentages are above 90% independent of P-CD concentration. The cell deaths of nematodes were insignificant even at high concentrations of P-CDs, such as 250 μg mL^−1^ for 48 h. This result advises the use of synthesized P-CDs for bio applications. 

Thus, P-CDs were further utilized as bioimaging agents. The nematode cells that are immobilized with 0.05% of sodium azide (NaN_3_) were incubated with 100 μg mL^−1^ P-CDs for 24 h and were observed under a confocal microscope with different filters. Blue, green, and red filters with a wavelength range of 400–470 nm, 470–550 nm and 550–620 nm, respectively, were used for the confocal microscope imaging. Cells were observed with excitation wavelengths of 400 (blue), 470 (green) and 550 (red) nm (Figure 7b–d). The confocal images demonstrate that the cells were coated with a uniform distribution of P-CDs. The uniform distribution of P-CDs over cells results in strong fluorescence intensity with a change in color, with a change in excitation wavelengths compared to the bright field (Figure 7e). The overlay image (Figure 7f) further confirms the uniform distribution of P-CDs over nematode cells.

## 4. Conclusions

In summary, we have successfully synthesized P-CDs from the PMPC polymer through a simple and cost-effective hydrothermal method. FESEM measurement reveals the uniform distribution of C, N, O and P in P-CDs. The HRTEM analysis of the synthesized P-CDs revealed a spherical shape with an average particle size of about 5 nm. Further, FTIR, XRD and XPS confirmed P-CDs enriched with functionalities and well graphitization. The optical studies revealed the excitation-dependent emission and stable fluorescence intensity of P-CDs. In addition, the P-CDs delivered an excellent quantum yield of 23%. Thus, P-CDs can be utilized as ink for hand drawing images, and handwriting texts, and as pads for tracing finger prints. The images, texts, and finger prints were stable for a long storage of about 4 months. The P-CDs showed low toxicity in the MTT assay. Thus, the prepared P-CDs was utilized as a bioimaging agent, and for the first time P-CDs in nematode cells with stable and strong fluorescence intensity were observed with blue, green and red emission. This study opens up new avenues for the development of bioimaging agents from polymers by hydrothermal treatment.

## Data Availability

Not applicable.

## Supplementary Materials

The following are available online at https://www.mdpi.com/article/10.3390/polym15061352/s1. Instrumentation methods, quantum yield measurement, photostability measurements, nematode killing assay, fluorescent staining, and imaging of the synthesized P-CDs, Figure S1. 1H-NMR (500 MHz) of PMPC in D_2_O; Figure S2. XRD pattern of synthesized P-CDs; Figure S3. (a) Raman spectrum and (b) deconvoluted with fitted Raman spectrum of synthesized P-CDs; Figure S4. XPS survey scan spectrum with atomic percentages of presented elements in the synthesized P-CDs; Figure S5. Enlarged UV–Vis absorption spectrum of the synthesized P-CDs; Figure S6. Fluorescence excitation-dependent emission normalized spectra of synthesized P-CDs.

## References

[1] Anand S.R., Bhati A., Saini D., Gunture Chauhan N., Khare P., Sonkar S.K. (2019). Antibacterial Nitrogen-doped Carbon Dots as a Reversible “Fluorescent Nanoswitch” and Fluorescent Ink. ACS Omega.

[2] Zhang X.D., Li J., Niu J.N., Bao X.P., Zhao H.D., Tan M. (2019). Fluorescent carbon dots derived from urine and their application for bio-imaging. Methods.

[3] Singh V., Rawat K.S., Mishra S., Baghel T., Fatima S., John A.A., Kalleti N., Singh D., Nazir A., Rath S.K. (2018). Biocompatible fluorescent carbon quantum dots prepared from beetroot extract for in vivo live imaging in C. elegans and BALB/c mice. J. Mater. Chem. B.

[4] Liu J., Li R., Yang B. (2020). Carbon Dots: A New Type of Carbon-Based Nanomaterial with Wide Applications. ACS Cent. Sci..

[5] Cui L., Ren X., Sun M., Liu H., Xia L. (2021). Carbon Dots: Synthesis, Properties and Applications. Nanomaterials.

[6] Dehvari K., Chiu S.-H., Lin J.-S., Girma W.M., Ling Y.-C., Chang J.-Y. (2020). Heteroatom doped carbon dots with nanoenzyme like properties as theranostic platforms for free radical scavenging, imaging, and chemotherapy. Acta Biomater..

[7] Supjaroenpisan M., Hanchaina R., Kangsamaksin T., Paoprasert P. (2021). Effects of Heteroatom Doping of Carbon Dots from Sugar on Optical Properties, Phenolic Content, Antioxidant Activity, Photostability, and Cytotoxicity. ChemistrySelect.

[8] Yao Y., Zhang H., Hu K., Nie G., Yang Y., Wang Y., Duan X., Wang S. (2022). Carbon dots based photocatalysis for environmental applications. J. Environ. Chem. Eng..

[9] Akbar E., Moretti A. (2021). Vomiero, Carbon Dots for Photocatalytic Degradation of Aqueous Pollutants: Recent Advancements. Adv. Opt. Mater..

[10] Saini D., Garg A.K., Dalal C., Anand S.R., Sonkar S.K., Sonker A.K., Westman G. (2022). Visible-Light-Promoted Photocatalytic Applications of Carbon Dots: A Review. ACS Appl. Nano Mater..

[11] Torres S., Bogireddy N.K., Kaur I., Batra V., Agarwal V. (2022). Heavy metal ion detection using green precursor derived carbon dots. iScience.

[12] Xu X.-J., Ge S., Li D.-Q., Xu Z.-Q., Wang E.-J., Wang S.-M. (2022). Fluorescent carbon dots for sensing metal ions and small molecules. Chin. J. Anal. Chem..

[13] Batool M., Junaid H.M., Tabassum S., Kanwal F., Abid K., Fatima Z., Shah A.T. (2022). Metal Ion Detection by Carbon Dots—A Review. Crit. Rev. Anal. Chem..

[14] Zhai Y., Zhang B., Shi R., Zhang S., Liu Y., Wang B., Zhang K., Waterhouse G.I., Zhang T., Lu S. (2022). Carbon Dots as New Building Blocks for Electrochemical Energy Storage and Electrocatalysis. Adv. Energy Mater..

[15] Siwal S.S., Kaur H., Saini A.K., Thakur V.K. (2022). Recent Progress in Carbon Dots-Based Materials for Electrochemical Energy Storage Toward Environmental Sustainability. Adv. Energy Sustain. Res..

[16] Wang B., Cai H., Waterhouse G.I.N., Qu X., Yang B., Lu S. (2022). Carbon Dots in Bioimaging, Biosensing and Therapeutics: A Comprehensive Review. Small Sci..

[17] Shen C.-L., Liu H.-R., Lou Q., Wang F., Liu K.-K., Dong L., Shan C.-X. (2022). Recent progress of carbon dots in targeted bioimaging and cancer therapy. Theranostics.

[18] Korah B.K., John N., John B.K., Mathew S., Bijimol D., Mathew B. (2022). Carbon dots as a fluorescent ink and dual-mode probe for the efficient detection of doxycycline and Hg(II) ions. J. Mater. Res..

[19] Al-Qahtani S.D., Hameed A., Snari R.M., Shah R., Alfi A.A., El-Metwaly N.M. (2022). Development of fluorescent carbon dots ink from rice straw waste toward security authentication. J. Mol. Liq..

[20] Kaczmarek A., Hoffman J., Morgiel J., Mościcki T., Stobiński L., Szymański Z., Małolepszy A. (2021). Luminescent Carbon Dots Synthesized by the Laser Ablation of Graphite in Polyethylenimine and Ethylenediamine. Materials.

[21] Ma Z., Ming H., Huang H., Liu Y., Kang Z. (2012). One-step ultrasonic synthesis of fluorescent N-doped carbon dots from glucose and their visible-light sensitive photocatalytic ability. New J. Chem..

[22] Li H., He X., Liu Y., Huang H., Lian S., Lee S.-T., Kang Z. (2011). One-step ultrasonic synthesis of water-soluble carbon nanoparticles with excellent photoluminescent properties. Carbon.

[23] Chao-Mujica F.J., Garcia-Hernández L., Camacho-López S., Camacho-López M., Camacho-López M.A., Reyes Contreras D., Pérez-Rodríguez A., Peña-Caravaca J.P., Páez-Rodríguez A., Darias-Gonzalez J.G. (2021). Carbon quantum dots by submerged arc discharge in water: Synthesis, characterization, and mechanism of formation. J. Appl. Phys..

[24] Ismail N.S., Husain U.S., Selvan S.I.S., Mordani N.A., Juhari N., Halim N.H.A. (2020). Effect of heating power towards synthesis of carbon dots through microwave pyrolysis method for optical-based biosensor. AIP Conf. Proc..

[25] Otten M., Hildebrandt M., Kühnemuth R., Karg M. (2022). Pyrolysis and Solvothermal Synthesis for Carbon Dots: Role of Purification and Molecular Fluorophores. Langmuir.

[26] Pang Z., Fu Y., Yu H., Liu S., Yu S., Liu Y., Wu Q., Liu Y., Nie G., Xu H. (2022). Efficient ethanol solvothermal synthesis of high-performance nitrogen-doped carbon quantum dots from lignin for metal ion nanosensing and cell imaging. Ind. Crops Prod..

[27] Kurian M., Paul A. (2021). Recent trends in the use of green sources for carbon dot synthesis–A short review. Carbon Trends.

[28] Ayiloor Rajesh G., John V.L., Pookunnath Santhosh A., Krishnan Nair Ambika A., Thavarool Puthiyedath V. (2022). Carbon Dots from Natural Sources for Biomedical Applications. Part. Part. Syst. Charact..

[29] Zhang H., Wang G., Zhang Z., Lei J.H., Liu T.-M., Xing G., Deng C.-X., Tang Z., Qu S. (2022). One step synthesis of efficient red emissive carbon dots and their bovine serum albumin composites with enhanced multi-photon fluorescence for in vivo bioimaging. Light Sci. Appl..

[30] Rabiee N., Iravani S., Varma R.S. (2022). Biowaste-Derived Carbon Dots: A Perspective on Biomedical Potentials. Molecules.

[31] Kang C., Huang Y., Yang H., Yan X.F., Chen Z.P. (2020). A Review of Carbon Dots Produced from Biomass Wastes. Nanomaterials.

[32] Yan H., Li P., Wen F., Xu Q., Guo Q., Su W. (2023). Green synthesis of carbon quantum dots from plant turmeric holds promise as novel photosensitizer for in vitro photodynamic antimicrobial activity. J. Mater. Res. Technol..

[33] Chahal S., Macairan J.-R., Yousefi N., Tufenkji N., Naccache R. (2021). Green synthesis of carbon dots and their applications. RSC Adv..

[34] Döring A., Ushakova E., Rogach A.L. (2022). Chiral carbon dots: Synthesis, optical properties, and emerging applications. Light Sci. Appl..

[35] Li M., Kuang Y., Fan Z., Qin X., Hu S., Liang Z., Liu Q., Zhang W., Wang B., Su Z. (2022). Simultaneous Electrochemical Sensing of Indole-3-Acetic Acid and Salicylic Acid on Poly(L-Proline) Nanoparticles-Carbon Dots-Multiwalled Carbon Nanotubes Composite-Modified Electrode. Sensors.

[36] Wang R., Gu W., Liu Z., Liu Y., Ma G., Wei J. (2021). Simple and Green Synthesis of Carbonized Polymer dots from Nylon 66 Waste Fibers and its Potential Application. ACS Omega.

[37] Lu D., Yin K., Hou S., Li P., Zhu H., Hao J., Li H. (2022). AlCl3-promoted growth of alkylated carbon dots with an enhanced nonlinear optical response. J. Mater. Chem. C.

[38] Yin K., Lu D., Tian W., Zhang R., Yu H., Gorecka E., Pociecha D., Godbert N., Hao J., Li H. (2020). Ordered structures of alkylated carbon dots and their applications in nonlinear optics. J. Mater. Chem. C.

[39] Lu D., Yin K., Zhao Y., Gao Z., Godbert N., Li H., Li H., Hao J. (2021). Facile synthesis of alkylated carbon dots with blue emission in halogenated benzene solvents. Colloids Surf. A Physicochem. Eng. Asp..

[40] Jorns M., Strickland S., Mullins M., Pappas D. (2022). Improved citric acid-derived carbon dots synthesis through microwave-based heating in a hydrothermal pressure vessel. RSC Adv..

[41] Paloncýová M., Langer M., Otyepka M. (2018). Structural Dynamics of Carbon Dots in Water and N, N-Dimethylformamide Probed by All-Atom Molecular Dynamics Simulations. J. Chem. Theory Comput..

[42] Xu A., Feng N., Yin K., Li H., Hao J. (2023). Supramolecular structures from structurally persistent and surface active carbon dots in water. Nanoscale.

[43] Siahcheshm P., Heiden P. (2023). High quantum yield carbon quantum dots as selective fluorescent turn-off probes for dual detection of Fe2+/Fe3+ ions. J. Photochem. Photobiol. A Chem..

[44] Tao S., Song Y., Zhu S., Shao J., Yang B. (2017). A new type of polymer carbon dots with high quantum yield: From synthesis to investigation on fluorescence mechanism. Polymer.

[45] Seetasang S., Xu Y. (2022). Recent progress and perspectives in applications of 2-methacryloyloxyethyl phosphorylcholine polymers in biodevices at small scales. J. Mater. Chem. B.

[46] Perumal S., Gangadaran P., Bae Y.W., Ahn B.C., Cheong I.W. (2021). Noncovalent Functionalized Graphene Nanocarriers from Graphite for Treating Thyroid Cancer Cells. ACS Biomater. Sci. Eng..

[47] Hu Q., Gong X., Liu L., Choi M.M.F. (2017). Characterization and Analytical Separation of Fluorescent Carbon Nanodots. J. Nanomater..

[48] Wu F., Su H., Wang K., Wong W.K., Zhu X. (2017). Facile synthesis of N-rich carbon quantum dots from porphyrins as efficient probes for bioimaging and biosensing in living cells. Int. J. Nanomed..

[49] Shaikh A.F., Tamboli M.S., Patil R.H., Bhan A., Ambekar J.D., Kale B.B. (2019). Bioinspired Carbon Quantum Dots: An Antibiofilm Agents. J. Nanosci. Nanotechnol..

[50] Rai S., Singh B.K., Bhartiya P., Singh A., Kumar H., Dutta P.K., Mehrotra G.K. (2017). Lignin derived reduced fluorescence carbon dots with theranostic approaches: Nano-drug-carrier and bioimaging. J. Lumin..

[51] Wu W., Zhan L., Ohkubo K., Yamada Y., Wu M., Fukuzumi S. (2015). Photocatalytic H2 evolution from NADH with carbon quantum dots/Pt and 2-phenyl-4-(1-naphthyl)quinolinium ion. J. Photochem. Photobiol. B.

[52] Edison T.N., Atchudan R., Sethuraman M.G., Shim J.J., Lee Y.R. (2016). Microwave assisted green synthesis of fluorescent N-doped carbon dots: Cytotoxicity and bio-imaging applications. J. Photochem. Photobiol. B Biol..

[53] Manimekalai K., Jayaprakash P. (2021). Influence of potassium chloride doping on the properties of potassium dihydrogen phosphate crystal for nonlinear optical applications. J. Mater. Sci. Mater. Electron..

[54] Hu Y., Geng X., Zhang L., Huang Z., Ge J., Li Z. (2017). Nitrogen-doped Carbon Dots Mediated Fluorescent on-off Assay for Rapid and Highly Sensitive Pyrophosphate and Alkaline Phosphatase Detection. Sci. Rep..

[55] Li L., Dong T. (2018). Photoluminescence tuning in carbon dots: Surface passivation or/and functionalization, heteroatom doping. J. Mater. Chem. C.

[56] Sengottuvelu D., Shaik A.K., Mishra S., Ahmad H., Abbaszadeh M., Hammer N.I., Kundu S. (2022). Multicolor Nitrogen-Doped Carbon Quantum Dots for Environment-Dependent Emission Tuning. ACS Omega.

[57] Ghosh S., Ghosh A., Ghosh G., Marjit K., Patra A. (2021). Deciphering the Relaxation Mechanism of Red-Emitting Carbon Dots Using Ultrafast Spectroscopy and Global Target Analysis. J. Phys. Chem. Lett..

[58] Zuo P., Chen Z., Yu F., Zhang J., Zuo W., Gao Y., Liu Q. (2020). An easy synthesis of nitrogen and phosphorus co-doped carbon dots as a probe for chloramphenicol. RSC Adv..

[59] Gong X., Lu W., Paau M.C., Hu Q., Wu X., Shuang S., Dong C., Choi M.M. (2015). Facile synthesis of nitrogen-doped carbon dots for Fe3+ sensing and cellular imaging. Anal. Chim. Acta.

[60] Shen R., Song K., Liu H., Li Y., Liu H. (2012). Dramatic fluorescence enhancement of bare carbon dots through facile reduction chemistry. Chemphyschem.

[61] Chen Y., Xiong G., Zhu L., Huang J., Chen X., Chen Y., Cao M. (2022). Enhanced Fluorescence and Environmental Stability of Red-Emissive Carbon Dots via Chemical Bonding with Cellulose Films. ACS Omega.

[62] Sharma A., Gadly T., Gupta A., Ballal A., Ghosh S.K., Kumbhakar M. (2016). Origin of Excitation Dependent Fluorescence in Carbon Nanodots. J. Phys. Chem. Lett..

[63] Zhou L., Wu F., Yu J., Deng Q., Zhang F., Wang G. (2017). Titanium carbide (Ti_3_C_2_Tx) MXene: A novel precursor to amphiphilic carbide-derived graphene quantum dots for fluorescent ink, light-emitting composite and bioimaging. Carbon.

[64] Holden M.A., Cremer P.S. (2003). Light Activated Patterning of Dye-Labeled Molecules on Surfaces. J. Am. Chem. Soc..

[65] Li B., Wei P., de Leon A., Frey T., Pentzer E. (2017). Polymer composites with photo-responsive phthalocyanine for patterning in color and fluorescence. Eur. Polym. J..

