# Supplementary material for: Water Soluble PMPC-Derived Bright Fluorescent Nitrogen/Phosphorous-Doped Carbon Dots for Fluorescent Ink (Anti-Counterfeiting) and Cellular Multicolor Imaging
Source: Polymers (Basel). 2023 Mar 8;15(6):1352. doi: 10.3390/polym15061352 (PMC10058053; doi:10.3390/polym15061352)
Supplement: Supplementary file 1 [file polymers-15-01352-s001.zip › polymers-2153260-supplementary.pdf]

Supplementary Materials

Water Soluble PMPC-Derived Bright Fluorescent Nitrogen/Phosphorous-Doped Carbon Dots for Fluorescent Ink (Anti-Counterfeiting) and Cellular Multicolor Imaging

Suguna Perumal [1,†], Raji Atchudan [2,3,*,†], Thomas Nesakumar Jebakumar Immanuel Edison [4,†], Sambasivam Sangaraju [5], Weslen Vedakumari Sathyaraj [6] and Yong Rok Lee [2,*]

[1] Department of Chemistry, Sejong University, Seoul 143747, Republic of Korea
[2] School of Chemical Engineering, Yeungnam University, Gyeongsan 38541, Republic of Korea
[3] Department of Chemistry, Saveetha School of Engineering, Saveetha Institute of Medical and Technical Sciences, Chennai-602105, Tamil Nadu, India
[4] Department of Chemistry, Sethu Institute of Technology, Kariapatti 626115, Virudhunagar District, Tamil Nadu, India
[5] National Water and Energy Center, United Arab Emirates University, Al Ain - 15551, United Arab Emirates
[6] Faculty of Allied Health Sciences, Chettinad Hospital and Research Institute, Chettinad Academy of Research and Education, Kelambakkam 603103, Tamil Nadu, India
* Correspondence: atchudanr@yu.ac.kr (R.A.); yrlee@yu.ac.kr (Y.R.L.)
† These authors contributed equally to this work.

Instrumentation Methods

The nuclear magnetic resonance (NMR) spectrum of the synthesized poly 2-(methacryloyloxy)ethyl phosphorylcholine (PMPC) was obtained using an NMR spectrometer, Bruker 500 MHz. A one-step hydrothermal-assisted carbonization process was adopted for synthesizing bright fluorescent nitrogen-doped carbon dots from poly 2-(methacryloyloxy)ethyl phosphorylcholine, and this polymer-derived carbon dots (P-CDs) was characterized by various physicochemical analytical techniques such as field emission scanning electron microscopy (FESEM) with energy-dispersive X-ray (EDX) spectroscopy, high-resolution transmittance electron microscopy (HRTEM), X-ray diffraction (XRD), Raman spectroscopy, attenuated total reflectance Fourier-transform infrared (ATR-FTIR) spectroscopy, X-ray photoelectron spectroscopy (XPS), Ultraviolet-visible (UV-vis) absorption spectroscopy, and fluorescence spectroscopy. FESEM with EDX analysis was carried out on a Hitachi S-

4800 equipped with EDX at an accelerating voltage of 10/15 kV. TEM/HRTEM images were performed with a JEOL JEM transmission electron microscope with an operating accelerating voltage of 120 kV. XRD measurements were carried out using a PANalytical X'Pert3 MRD diffractometer with monochromatized Cu Kα radiation (λ = 1.54 Å) at 40 kV and 30 mA and were recorded in the range from 10 to 80° (2θ). Raman spectrum was recorded on XploRA Micro-Raman spectrophotometer (Horiba) with ranges between 50 and 4000 cm^{-1} at the core research support center for natural products and medical materials of Yeungnam University. ATR-FTIR spectra were recorded in transmittance mode on a Perkin Elmer Spectrum Two in the wavenumber range from 400 to 4000 cm^{-1} by the addition of 8 scans at a resolution of 8 cm^{-1}. XPS spectra were achieved using a K-Alpha (Thermo Scientific). CasaXPS software was used for the deconvolution of the high-resolution XPS spectra. UV-vis absorption spectra were recorded from 200 to 700 nm using an OPTIZEN 3220UV spectrophotometer. Excitation and emission fluorescence spectra were recorded using a Hitachi F-7000 fluorescence spectrophotometer using a 1 cm^3 path-length quartz cell. The excitation wavelength was varied to determine the maximum emission intensity. The slit width was fixed at 5 nm, and the scan speed was set to 400 nm/min.

Quantum Yield Measurement of P-CDs

The quantum yield (QY) of the synthesized P-CDs was calculated by using quinine sulfate in 0.1 M H_2SO_4 (QY_R is 0.54) as a standard reference and was calculated using the following equation (S1):

$$QY = QY_R \frac{I_S A_R (n_S)^2}{I_R A_S (n_R)^2} \qquad \text{(S1)}$$

where, "I" is the measured integrated fluorescent emission intensity, "n" is the refractive index of the solvent, and "A" is the absorbance (intensity). The subscript "R" and "S" refer to the known fluorescent reference and standard for the synthesized sample, respectively.

Photostability Measurements of P-CDs

The photostability of the synthesized P-CDs was examined by continuous irradiation under UV light (365 nm) for 150 min with an interval of every 50 min. The fluorescence intensity of the P-CDs aqueous solution was measured every 50 min until 150 min. The fluorescence intensity of the P-CDs aqueous solution after 150 min UV-light irradiation was compared to the initial (0 min) one to determine their anti-

photobleaching ability (photostability).

Nematode Killing Assay

Toxicity analysis of the lead compound was performed for this Caenorhabditis elegans/nematodes (approximately 60 nematodes per well of 98 well non-binding plates, Corning™, USA) were counted and suspended in S-basal buffer. Each well plate was then treated with various concentrations of P-CDs (0, 50, 100, 150, 200, and 250 μg mL^{-1}), following which the plates were incubated at 25 °C for 48 h. After incubation, the nematodes were scrutinized and observed for dead nematodes by prodding with a platinum wire gauge. The viability value (%) of nematodes was calculated using equation (S2). All experiments were performed in three replicates.

$$\text{Nematodes Viability (\%)} = \frac{N_{(T)} - N_{(D)}}{N_{(T)}} x100 \qquad \text{(S2)}$$

where $N_{(T)}$ and $N_{(D)}$ are the total number of nematodes and dead nematodes, respectively.

Fluorescent Staining and Imaging

Staining and imaging assays were done as follows; the live nematodes in the S-basal buffer were collected and centrifuged at 10,000 rpm for 5 min. The supernatant was removed, and the nematode pellet was stained with P-CDs (100 μg mL^{-1}) and incubated for 24 h at room temperature (25 °C). The excess staining solution (P-CDs) was removed by further centrifugation, and the nematodes were resuspended in an S-basal buffer and imaged under a fluorescent microscope at 10X magnification.

Characterization of PMPC

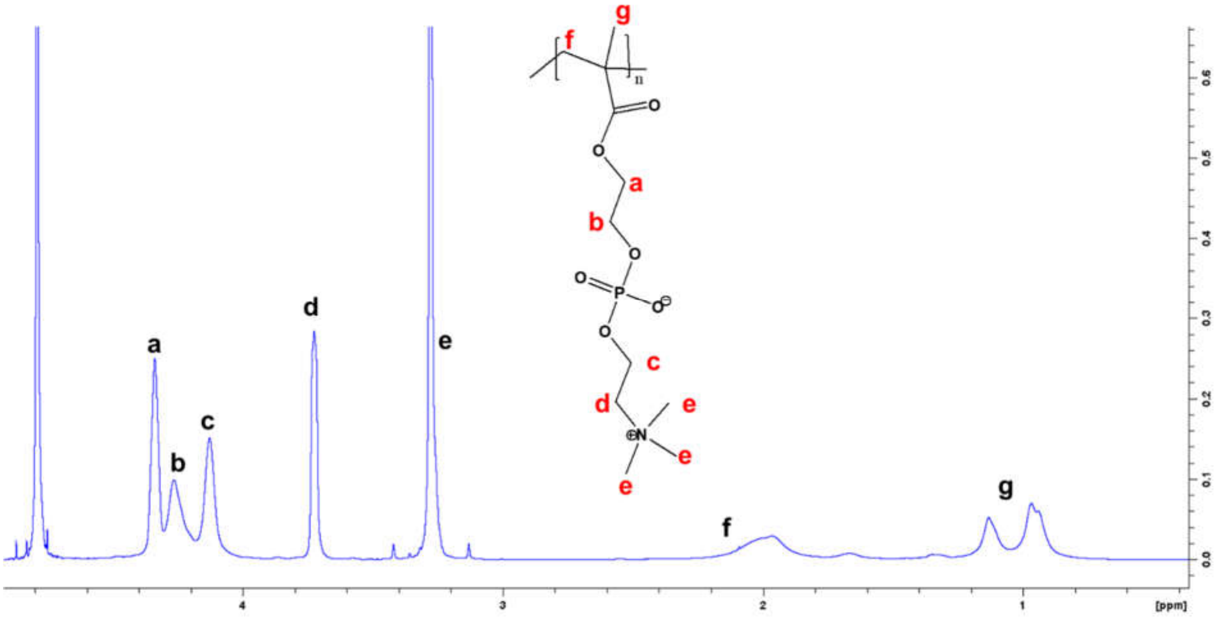

Figure S1. ^{1}H-NMR (500 MHz) of PMPC in D_2O.

Structural Properties of Synthesized P-CDs

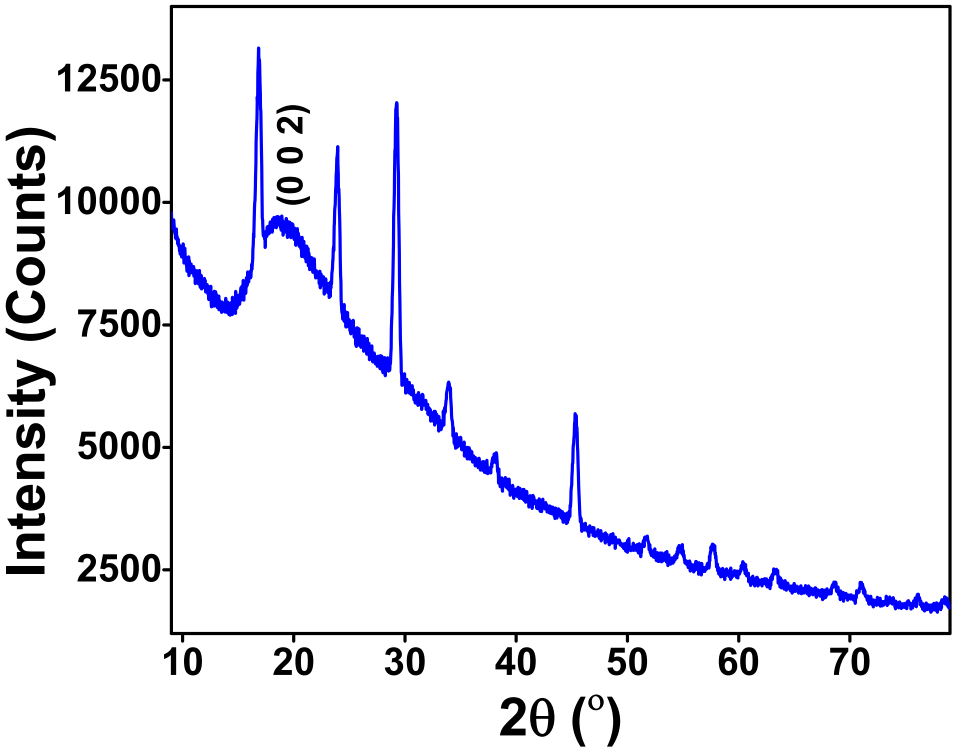

Figure S2. XRD pattern of synthesized P-CDs.

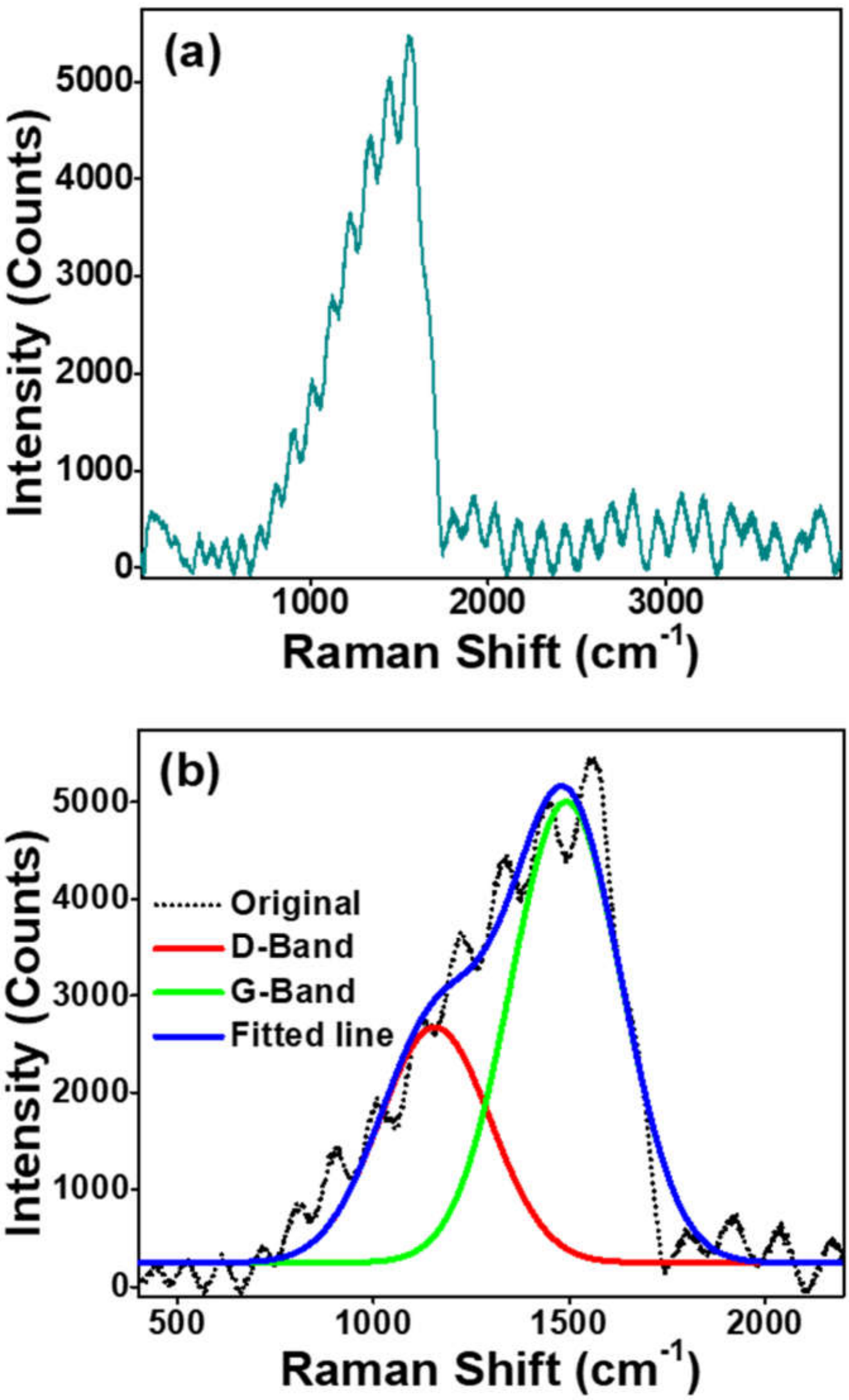

Figure S3. (a) Raman spectrum and (b) deconvoluted with fitted Raman spectrum of synthesized P-CDs.

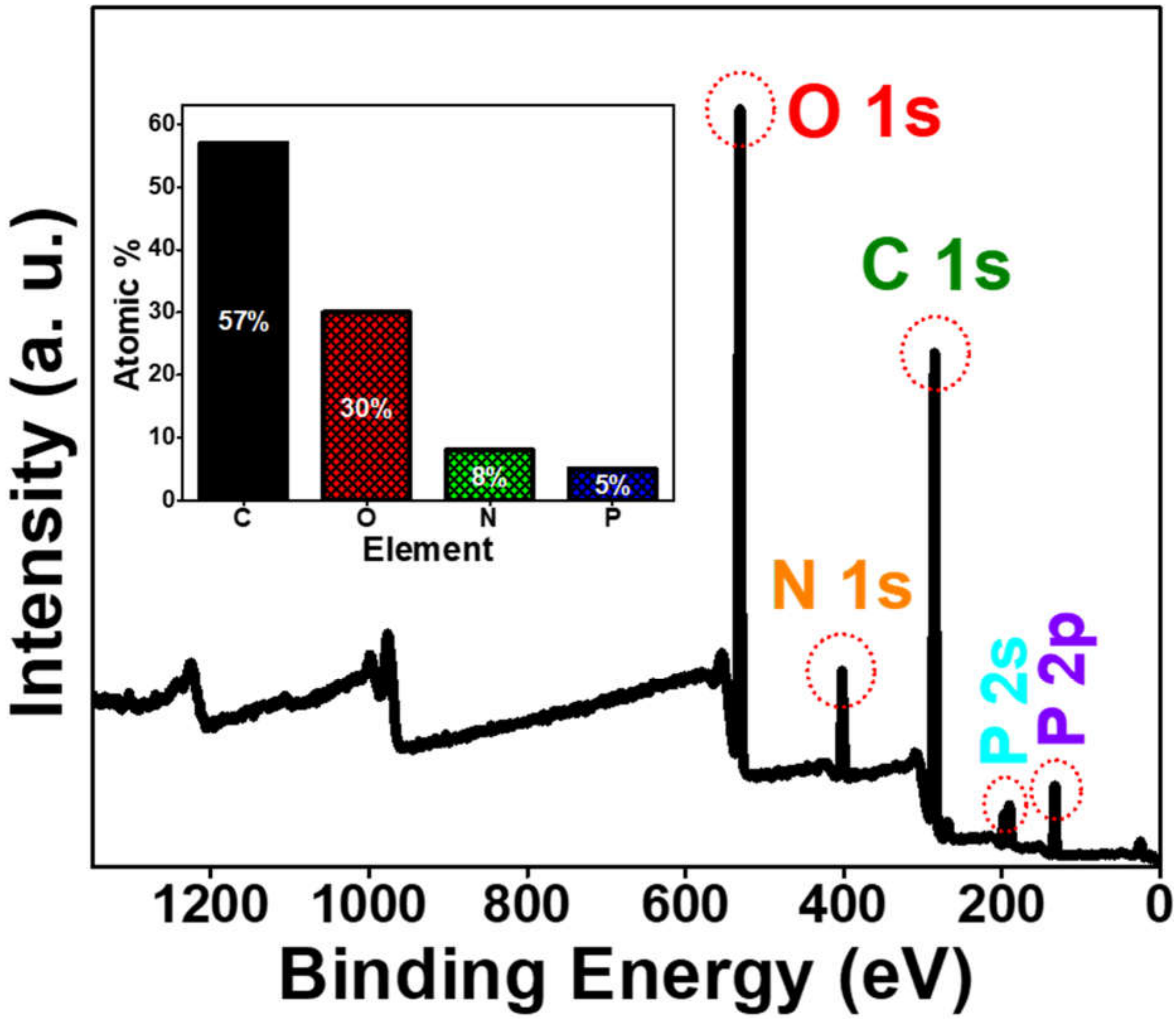

Figure S4. XPS survey scan spectrum with atomic percentages of presented elements in the synthesized P-CDs.

Optical Properties of Synthesized P-CDs

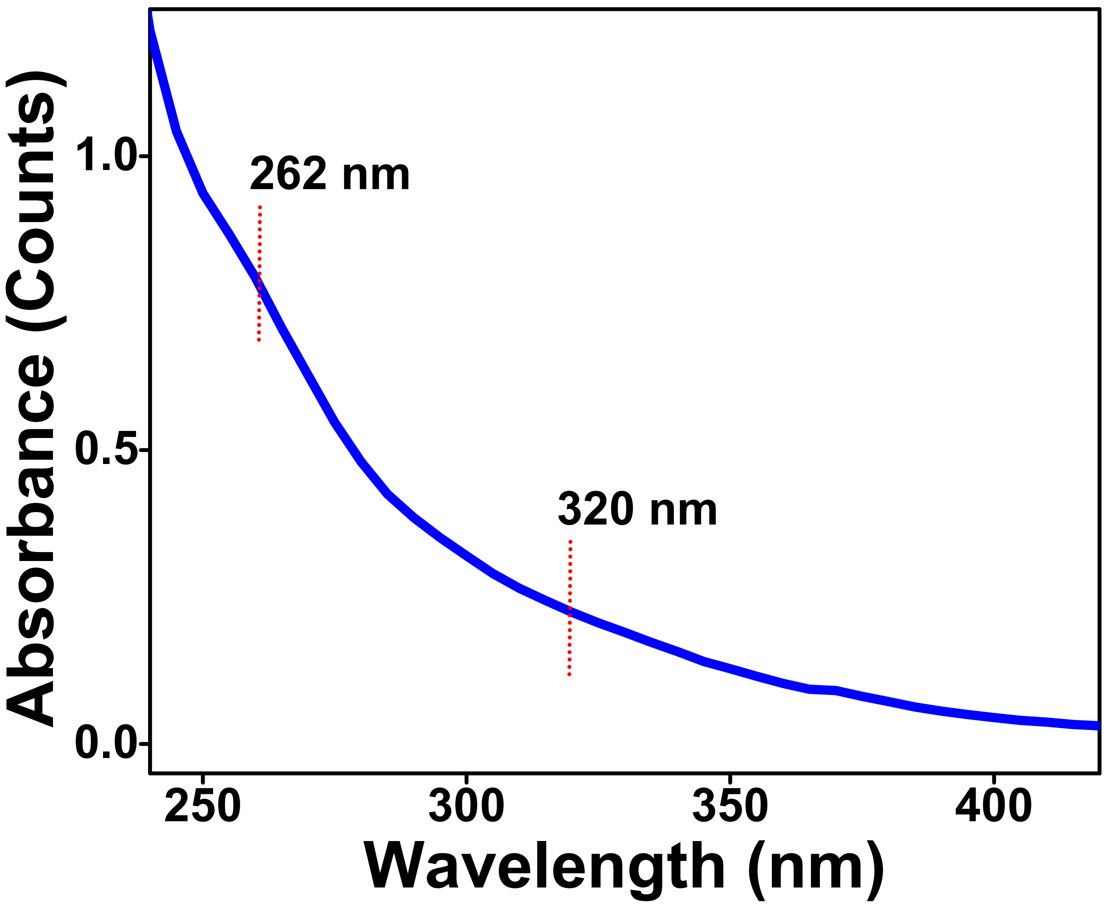

Figure S5. Enlarged UV–Vis absorption spectrum of the synthesized P-CDs.

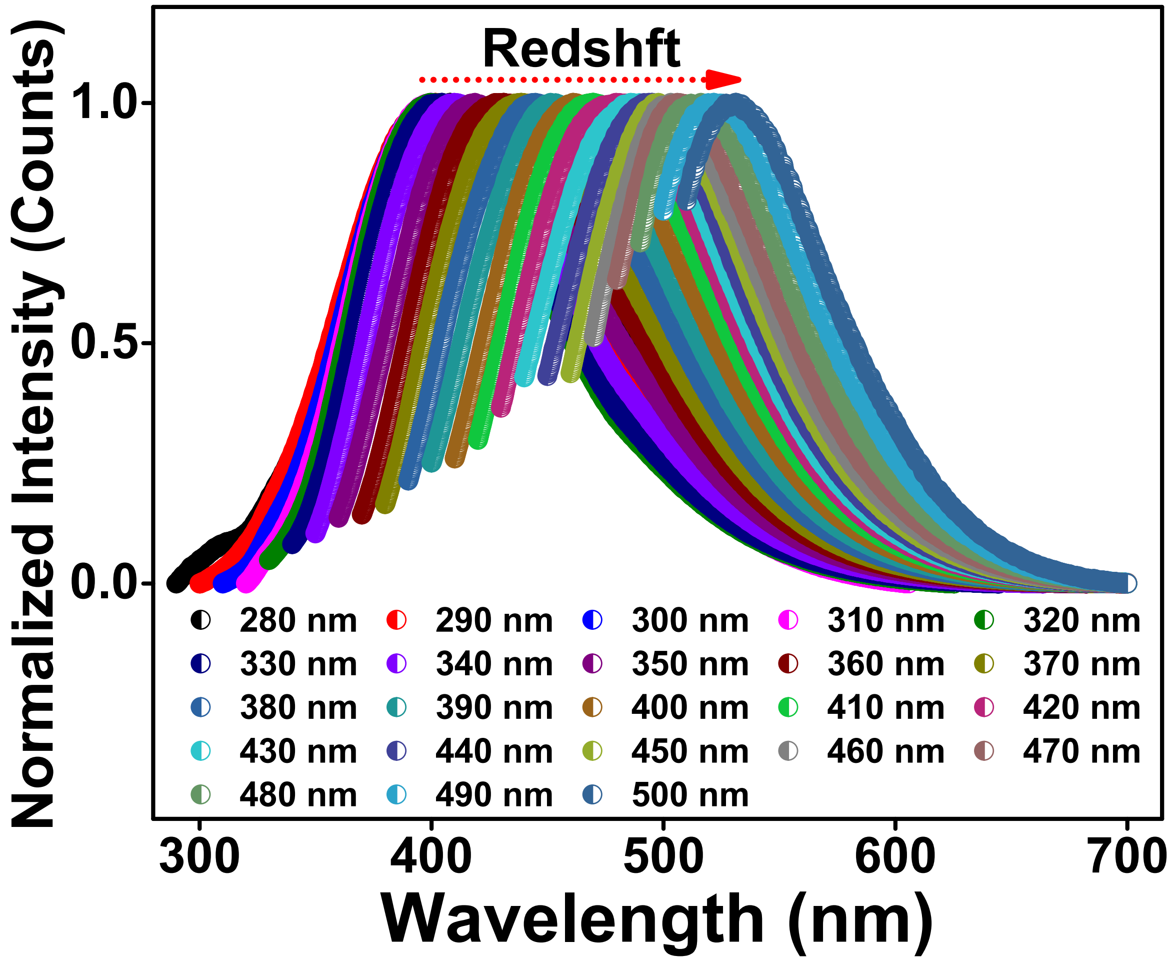

Figure S6. Fluorescence excitation-dependent emission normalized spectra of synthesized P-CDs.
